# Comparative genomic analysis of *Methanimicrococcus blatticola* provides insights into host adaptation in archaea and the evolution of methanogenesis

**DOI:** 10.1038/s43705-021-00050-y

**Published:** 2021-09-09

**Authors:** Courtney M. Thomas, Najwa Taib, Simonetta Gribaldo, Guillaume Borrel

**Affiliations:** 1 Department of Microbiology, UMR 2001, Unit Evolutionary Biology of the Microbial Cell, Institut Pasteur, Paris, France; 2 Université Paris Diderot, Sorbonne Paris Cité, Paris, France

**Keywords:** Archaeal genomics, Phylogenetics, Metabolism

## Abstract

Other than the Methanobacteriales and Methanomassiliicoccales, the characteristics of archaea that inhabit the animal microbiome are largely unknown. *Methanimicrococcus blatticola*, a member of the Methanosarcinales, currently reunites two unique features within this order: it is a colonizer of the animal digestive tract and can only reduce methyl compounds with H_2_ for methanogenesis, a increasingly recognized metabolism in the archaea and whose origin remains debated. To understand the origin of these characteristics, we have carried out a large-scale comparative genomic analysis. We infer the loss of more than a thousand genes in *M. blatticola*, by far the largest genome reduction across all Methanosarcinales. These include numerous elements for sensing the environment and adapting to more stable gut conditions, as well as a significant remodeling of the cell surface components likely involved in host and gut microbiota interactions. Several of these modifications parallel those previously observed in phylogenetically distant archaea and bacteria from the animal microbiome, suggesting large-scale convergent mechanisms of adaptation to the gut. Strikingly, *M. blatticola* has lost almost all genes coding for the H_4_MPT methyl branch of the Wood–Ljungdahl pathway (to the exception of *mer*), a phenomenon never reported before in any member of Class I or Class II methanogens. The loss of this pathway illustrates one of the evolutionary processes that may have led to the emergence of methyl-reducing hydrogenotrophic methanogens, possibly linked to the colonization of organic-rich environments (including the animal gut) where both methyl compounds and hydrogen are abundant.

## Introduction

Methanogenic archaea are common components of the intestinal microbiota of animals ranging from insects to humans [1–4]. However, archaea are generally overlooked in intestinal microbiome studies, leaving their ecology and diversity largely undescribed in this context. Additionally, only a small number of gut-associated archaeal isolates exists outside of the *Methanobrevibacter* and *Methanosphaera* genera (both belonging to the order Methanobacteriales), resulting in little knowledge concerning the adaptations that allowed archaea to colonize the animal gut. The physical/chemical conditions of a microbe’s niche exert selective pressures that can influence its genome content [5]. The gut of animals is characterized by a high and almost constant intake of fresh organic matter that distinguishes it from most aquatic and terrestrial environments. At the same time, gut microbes have to adapt to stressors such as potential removal from the host by the peristaltic movement of the digestive tract [5]. Host-associated archaea lineages have not only distinct genetic background due to their distant evolutionary relationships but also different histories of adaptation to the gut, and they can cover a wide variety of hosts. This poses the question of whether these distantly related archaea may display convergent adaptations or not. Genomic adaptations to the intestinal microbiome have been proposed in some methanogens: these include the gain of genes coding for cell surface proteins facilitating interactions or adhesion and possibly genomic streamlining [3, 6–10].

Within the order Methanosarcinales, two host-associated methanogens have been isolated, *Methanimicrococcus blatticola* PA from a cockroach (*Periplaneta americana*) [11] and *Methanosarcina barkeri* CM1 from the rumen of a cow [12]. However, the genome of *M. barkeri* CM1 is very similar to that of its freshwater close relative *M. barkeri* fusaro [13] and *Methanosarcina* members do not regularly occur in the rumen microbiota, suggesting a generalist lifestyle or a transient presence in the gut. In contrast, *Methanimicrococcus* has been reported multiple times in the gut of animals, and in some cases, it was found to represent a large majority of the methanogenic community in termites and some ruminants [14, 15]. Based on this apparent niche specificity, *M. blatticola* should display specific genomic adaptations to a host-associated lifestyle.

In addition to being an interesting model to study adaptations of archaea to the gut environment, *M. blatticola* is the only known member of Methanosarcinales that obligately uses H_2_ to reduce methyl compounds [11, 16, 17] (i.e., methyl-reducing hydrogenotrophic methanogenesis), a metabolism that has been recently reported in a growingly large number of newly discovered methanogen lineages [18–23]. Here we have analyzed the genome of *M. blatticola* PA, currently the only available isolated Methanimicrococcus strain, and carried out a large-scale comparative genomic analysis to understand the emergence of its unique characteristics. Our results provide new insights into the processes leading to the adaptation of archaea to the animal digestive tract and highlight one of the possible paths that led to the emergence of methyl-reducing hydrogenotrophic methanogenesis.

## Materials and methods

### Genome sequencing and annotation

The *M. blatticola* PA genome was sequenced from DNA ordered at the DSMZ German culture collection (DSM 13328), using Illumina MiSeq Nano V2 (2 ×250 PE). Reads were assembled using Spades 3.11 [24]. A total of 12 contigs >1 kb were obtained, with an average coverage of 157×. Almost all reads were assembled, as 99.97% of them aligned on these contigs. Genes were predicted using prodigal [25]. The genome and protein sequences are available in GenBank, PRJNA731512, and in Supplementary Dataset 1 and 2. Gene functions were annotated with Kyoto Encyclopedia of Genes and Genomes (KEGG) [26] and Eggnog [27]. Genes associated with the methanogenesis pathways were specifically targeted with HMM searches using PFAM [28], TIGRFAMs [29], and custom HMM profiles. Presence of transmembrane domains was determined using TMHMM 2.0 [30]. In the course of our analysis, this strain was also sequenced by JGI (2756170388) and released in GenBank (GCA_004363215.1). Unsurprisingly, the sequences and statistics of the two genomes are practically identical (size difference of <1 kb, average nucleotide identity (ANI) of 99.99%, 13 contigs). The analyses presented here were conducted on the genome we sequenced.

### Dataset selection

Phylogenetic and comparative genomic analyses were carried out using 21 Methanosarcinales genomes, including 15 *Methanosarcinaceae* (Table S1). We prioritized the selection of high-quality genomes, a majority of which came from cultured and well-characterized species. Genomes from metagenomes were only included when no genome from an isolated strain was available and only if the estimated completeness level was >90% and the contamination <5%, as estimated with CheckM [31]. Four additional genomes were used as outgroups, three Methanocellales and one “*Ca*. Methanoflorentaceae” genomes, corresponding to close relatives to the Methanosarcinales.

### Glycosyltransferase (GT) and PGF-CTERM annotation

GTs were annotated using dbCAN2 [32]. dbCAN2 was run on 507 archaeal genomes from a local database (Table S2), covering all major lineages of archaea and having >75% of completeness and <5% contamination. GTs in Methanosarcinales of the selected genome dataset described above was determined using the annotation found in the CAZy database [33].

Proteins containing a PGF-CTERM domain, which are associated with the PGF-CTERM/archaeosortase A system and possibly N-glycosylated, were first identified in the 21 Methanosarcinales plus the *M. blatticola* PA genomes using TIGR04126 HMM profile. Because this domain is short, new HMM profiles were generated from the PGF-CTERM domains of Methanosarcinales and *Methanimicrococcus* proteins (Supplementary Dataset 3). These new HMM profiles allowed the identification of extra proteins with a PGF-CTERM domain.

### Phylogenetic analyses

The phylogenetic position of *M. blatticola* PA was established using three different datasets: (i) the concatenation of 40 universal markers as described in [18] that correspond to 36 proteins of the Phylosift dataset [34], plus the alpha and beta subunits of the RNA polymerase and two universal ribosomal proteins (L30, S4), (ii) the concatenation of the three MCR subunits (McrABG), and (iii) the 16S rRNA gene. The protein sequences for the phylogenetic analysis were retrieved from *M. blatticola* PA and the other 25 selected genomes using HMMer [35]. Single-protein datasets were aligned using MAFFT [36] (mafft-linsi), trimmed with BMGE [37] (BLOSUM30 substitution matrix) and concatenated. The 16S rRNA genes of *M. blatticola* PA and the 25 selected genomes were also aligned with MAFFT. A Bayesian phylogeny was built for the concatenation of the 40 universal markers using PhyloBayes [38] under the CAT + GTR + Γ4 model with four independent MMC chains, until convergence (maxdiff < 0.05). For the concatenation of the McrABG and the 16S rRNA gene sequences, Maximum Likelihood phylogenies were calculated with IQ-Tree [39] with the NEWTEST option for best model selection (McrABG: LG + F + Γ4; 16S: GTR + R3) and 1000 ultrafast bootstrap iterations. To investigate the environmental distribution of *Methanimicrococcus*, 16S rRNA gene sequences were searched in GenBank (Nucleotide collection nr/nt) and in all assembled metagenomes of the IMG databases using BLAST. Sequences >750 bp and with >91% identity with *M. blatticola* PA were downloaded. This identity cutoff was based on the minimal distance between *M. blatticola* PA and Methanosarcina spp. 16S rRNA gene sequences. A phylogeny of these sequences was built as described above for the 16S rRNA genes. Fasta files of the sequences used for phylogeny are available in Supplementary Dataset 4.

### Gene flow reconstruction

This analysis was based on the phylogeny of the 40 concatenated markers described above and on the distribution of protein families among the 26 taxa used in this phylogeny. The protein families were determined using Silix with a 40% identity and 80% coverage cut-off [40], generating 21,377 protein families whose distribution was mapped on the reference phylogeny. The events of gene gains and losses that occurred during the evolution of the Methanosarcinales, and in particular on the branch leading to *M. blatticola* PA, were assessed using a death and birth model implemented in Count [41]. The rate of variation across families were optimized iteratively from uniform rates of gain, loss, and duplication to three discrete categories for the gamma distribution, using default parameters. Family history was calculated using posterior probabilities. *M. blatticola* PA protein sequences and representative of *Methanosarcinaceae* protein families that have been lost in *M. blatticola* PA were compared with three recently released Methanimicrococcus metagenome-assembled genomes (MAGs; i.e., GCA_009784005.1, GCA_009783635.1, and GCA_009776675.1, having 92.1/0%, 82.5/2.3%, 75.1/4.6% of completeness/contamination, respectively, according to CheckM [31], Table S3). A 40% identity and 50% coverage cut-off was used to determine whether proteins present or predicted to be lost in *M. blatticola* PA were coded in these MAGs. A lower coverage cut-off than in the main analysis was chosen because two of these MAGs are sheared (138 and 239 contigs), and as a consequence, a number of their proteins can be cut and split between different contigs.

## Results and discussion

### Widespread association of the *Methanimicrococcus* genus with gut microbiomes and extensive genome reduction in *M. blatticola*

We sequenced the genome of *M. blatticola* PA from the DSMZ German culture collection (DSM 13328). The draft genome (12 contigs) is 1.78 Mb long with a GC content of 42% and is predicted to encode 1569 proteins (Table S1). It is practically identical to the one sequenced by JGI (2756170388) in the course of our analysis and released in GenBank (GCA_004363215.1) (size difference of <1 kb, ANI of 99.99%, 13 contigs). The analyses presented hereafter were conducted on the genome we sequenced.

**Fig. 1:**
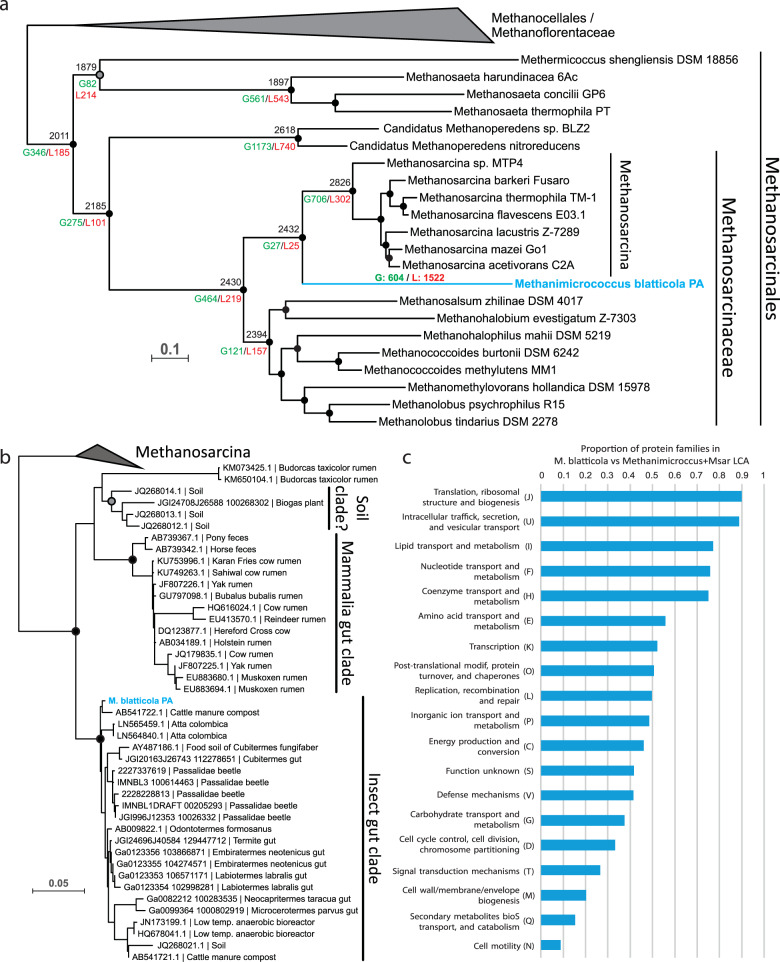
Phylogenetic position, gene flow, and environmental distribution of *Methanimicrococcus*. **a** Bayesian phylogeny (PhyloBayes, CAT + GTR + Γ4) of the Methanosarcinales based on a concatenation of 40 markers universal markers (10,126 positions). Numbers on the nodes indicate the genes present (black), gained (green, preceded by “G”), and lost (red preceded by “L”) along the evolution of the Methanosarcinales, as predicted by Count program [41]. The color of the circles at the nodes indicates the posterior probability values (black, >0.95; gray, between 0.70 and 0.95). **b** Maximum likelihood phylogeny (GTR + I + Γ4) based on the 16S rRNA gene sequences (1497 positions), displaying the environments where *Methanimicrococcus* occurs, mainly insect and mammalian digestive tract. The soil clade is followed by a question mark as all sequences of this poorly supported clade come from a single unpublished study, and their origin is therefore not totally reliable. The color of the circles at the nodes indicates ultrafast bootstrap values (black, >95; gray, between 70 and 95). The supports of the branches are only displayed for the main clades. **c** For each COG category, proportion of protein families in *Methanimicrococcus blatticola* PA relative to those present in the last common ancestor (LCA) of *Methanimicrococcus* + *Methanosarcina* (Msar).

We first established the phylogenetic position of *M. blatticola* PA using three different datasets: the concatenation of 40 universal markers as described in [18] (Fig. 1a), the 16S rRNA gene (Fig. S1), and a concatenation of the three MCR subunits (McrABG; Fig. S2). In all three resulting phylogenies, *M. blatticola* PA consistently and robustly branches within *Methanosarcinaceae*, at the base of *Methanosarcina*. This placement is different from the one previously proposed, at the base of the *Methanosarcinaceae*, but the phylogeny was poorly resolved [11].

Investigation of the environmental distribution of *Methanimicrococcus* members based on 16S rRNA gene sequences retrieved from GenBank and IMG databases revealed that they commonly occur in the animal digestive tract (Fig. 1b), in contrast to other members of the Methanosarcinales that are generally found in wetland soils and sediments [42]. Three distinct clades can be observed within the *Methanimicrococcus* genus: one is formed by sequences mainly from the digestive tract of insects (including *M. blatticola* PA), the second corresponds to sequences from mammalian digestive tracts, and the third consists of a few sequences from a single unpublished study on soil (Fig. 1b). These three clusters may indicate either one ancestral or two independent specializations to the animal digestive tract (mammals and insects) in *Methanimicrococcus*.

To investigate how genome content was affected during the transition from an open environment to the animal gut, we inferred the gene gains and losses that occurred between the last common ancestor (LCA) of *Methanosarcina*/*Methanimicrococcus* and *M. blatticola* PA. This analysis revealed an important shift in the gene content (Fig. 1a and Table S4). Indeed, one-third of *M. blatticola* PA genes (604 genes) were not present in *Methanosarcina*/*Methanimicrococcus* LCA (Fig. 1a) whereas two-thirds of the genes present in *Methanosarcina*/*Methanimicrococcus* LCA were lost (1522 genes), corresponding by far to the largest genome reduction across all Methanosarcinales (Fig. 1a). The small number of proteins encoded in the *M. blatticola* genome (1569) compared to *Methanosarcina* (3400 in average) or most other *Methanosarcinaceae* (2300 in average) further supports this prediction (Table S1). All Clusters of Orthologous Genes (COG) categories display a net loss in *M. blatticola* PA compared to *Methanosarcina/Methanimicrococcus* LCA (Fig. 1c) but some are more impacted than others. Several core cellular processes, such as “Translation, ribosomal structure and biogenesis (J),” “Lipid transport and metabolism (I),” or “Nucleotide transport and metabolism (F)” are only weakly affected, while others, such as cell envelope biogenesis, display a massive reduction in the number of protein families involved (Fig. 1c). A majority of the acquired genes have only a general or no predicted function (60% have no COG category and 80% no KEGG annotation), in contrast with those that were lost (4% have no COG category and 57% no KEGG annotation; Table S4).

### Extensive modification of the cell–host interaction surface

An expected trait of adaptation to a host-associated lifestyle is modification of the repertoire of membrane-bound proteins, situated at the interface between the cell and its environment. Indeed, we observed that two-thirds of the proteins inferred to be membrane bound in *M. blatticola* PA were not present in *Methanosarcina*/*Methanimicrococcus* LCA and were therefore specifically acquired in the lineage leading to this archaeon. Similar to the cytoplasmic proteins acquired by *M. blatticola* PA, most of the membrane-associated proteins have an unknown function (Table S4). However, among them, several correspond to putative adhesin-like proteins (ALPs) (Tables S4 and 1). They are annotated as cell wall-binding repeat-containing protein or collagen-binding protein, and two contain collagen or cellulose (CBM44) binding domains. Sixteen acquired proteins contain one or several Listeria-Bacteroidetes repeats/Flg_New (PF09479) domains that display structural similarities with β-grasp folds, having various binding functions [43]. In Archaea, proteins with these domains were previously only known from Methanomassiliicoccales of the human gut (up to 38 in “*Ca*. Methanomassiliicoccus intestinalis”) where they have been suggested to be involved in the attachment to specific sites in the digestive tract [3]. Bacteria with a high number of genes coding for Listeria-Bacteroidetes repeats/Flg_New domains are also associated with an animal host [3]. The presence of these putative ALPs in two phylogenetically distant gut-associated archaeal lineages (belonging to Methanomassiliicoccales and Methanosarcinales) and in host-associated bacteria further supports the hypothesis that they are specific adaptations to the digestive tract [3] and indicate convergent adaptation. Among the acquired genes coding for membrane-bound proteins, 15 contain a GLUG motif of unknown function (2 of them also have Flg_new domains) and may also have a role in adhesion. In the absence of flagella, these proteins may prevent the washout of *M. blatticola* PA cells from the insect gut and help them to remain at specific sites where they are most competitive for substrates [14]. It was shown in fact that many methanogens associated with cockroaches are loosely associated with chitin bristles present in the hindgut [1].

**Table 1:** Summary of the genes gained and lost in *M. blatticola* PA, discussed in the text as adaptations to a host-associated lifestyle, and comparison with the presence/absence patterns in other host-associated archaea and bacteria reported in the literature.

Genes	State	Function	Similar pres/abs patterns in 3 *Methanimicrococcus* MAGs^a^	Similar pres/abs patterns in other host-associated archaea^b^	Similar to gut-associated bacteria^c^
*fwdABCDEFG*, *fmdBD*, *ftr*, *mch*, *mtd*, *mfnBDEF*, *moaABC/mobAB/moeA,* modABCDE, wtpAB, *tupAB*, *Rnf*, *Fpo-like*	Lost	Methylotrophic and CO_2_-reducing methanogenesis	Yes	*moaABC*/*moeA* in *Methanosphaera*	
*comDE*	Lost	Coenzyme-M biosynthesis	Yes	“*Ca*. Methanoplasma termitum”, ISO4-H4, ISO4-G1, *Methanobrevibacter ruminantium*	
*pstABC*	Lost	High-affinity phosphate transporter	Absent in GCA009776675		
*phoA*	Lost	Organo-phosphate utilization	Yes		
*nifKDU*	Lost	N_2_ fixation	Yes	*Methanobrevibacter*	
*cdhAB*	Lost	CO_2_ fixation	Yes	*Methanobrevibacter*	
*asnB*, *trpABDFGE*	Lost	Amino acid synthesis	Most *trp* genes present in GCA009776675		
*cstA*	Gained	Uptake of pyruvate and peptides	No	“*Ca*. Methanomassiliicoccus intestinalis”	
m19, m21, m24	Lost	MCR-associated markers; regulatory processes of methane metabolism related to changes in substrate/nutrient availability^d^	Yes	Host-associated Methanomassiliicoccales, *Methanobrevibacter*, *Methanosphaera*	
*gys*, *gyp*, *PK_C*, *pfkC*, *galU*	Lost	Glycogen synthesis and degradation	Yes		
*cheABCFDRWY*	Lost	Sensing environment	Yes		Yes
19 histidine kinase/PAS sensor	Lost	Sensing environment	Yes (18/19 also absent in the three MAGs)		Yes
*flaBCEFGH*	Lost	Motility	Yes		Yes
43 transcriptional regulators	Lost	Modification of gene expression	Yes (37/43 also absent in the three MAGs)	*Methanobrevibacter smithii*, *Methanosphaera stadtmanae*	Yes
16 genes encoding a Flg_new domain	Gained	Adhesion^d^	Yes (3 to 13 present)	Host-associated Methanomassiliicoccales	Yes
MBLAb0210	Gained	Binding to cellulose (CBM44)	Yes		
MBLAb0400	Gained	Binding to pectins^d^	Yes		
MBLAb1410	Gained	Binding to cell wall^d^ (CBM37)	No		
MBLAb1461	Gained	Binding to collagen^d^	No		
MBLAb1469	Gained	Cell wall-binding repeat	No		
*glk*, *rfbA*, *rfbB*, *rfbC*, *rfbD*, *galU*, *galE*, *ugd*, *glmS*, *glmU*, *wecB*, *wecC*, *wbpP*, *aglA/manAC*, *gmd*, *flc*	Lost	Synthesis of nucleotide-activated sugars, precursors of extracellular polysaccharides	Yes		
35 glycosyltransferases	Lost	Cell surface modification	Yes	Host-associated Methanomassiliicoccales, *Methanobrevibacter*	
*artB*	Lost	VPXXXP-CTERM-specific archaeosortases	Yes		
*artC*	Lost	PEF-CTERM-specific archaeosortases	Yes		

*pres/abs* presence/absence.

^a^Similar pattern means that gene lost in *M. blatticola* PA are absent in the three *Methanimicrococcus* MAGs or that genes gained in *M. blatticola* PA are present in these MAGs.

^b^Lineages indicated in this column have patterns of gene presence/absence (from literature cited in the main text) that match the gene gains and losses reported in *M. blatticola* PA.

^c^See literature cited in the main text.

^d^Putative function.

**Fig. 2:**
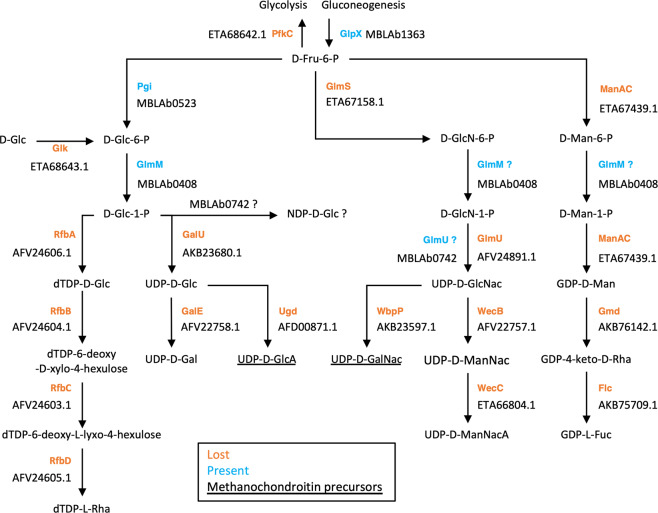
Loss of multiple enzymes involved in the synthesis of nucleotide-activated sugars that are precursors of cell-surface glycoconjugates/extracellular polysaccharides. The enzyme names in orange indicate that the corresponding genes were lost in *M. blatticola* PA, while those in blue correspond to genes that have been kept in *M. blatticola* PA. The genes lost in *M. blatticola* PA are also absent in the three *Methanimicrococcus* MAGs and those present in *M. blatticola* PA are also present in the three *Methanimicrococcus* MAGs. In other organisms, GlmM could perform three different reactions, albeit with different level of activity. Methanosarcinales genomes have two copies of *glmM*, but *M. blatticola* PA has only one. This enzyme is likely involved in the conversion of glucose-6-P into glucose-1-P. The accession numbers below the protein names provide an example of sequences in the *Methanosarcinaceae*.

**Fig. 3:**
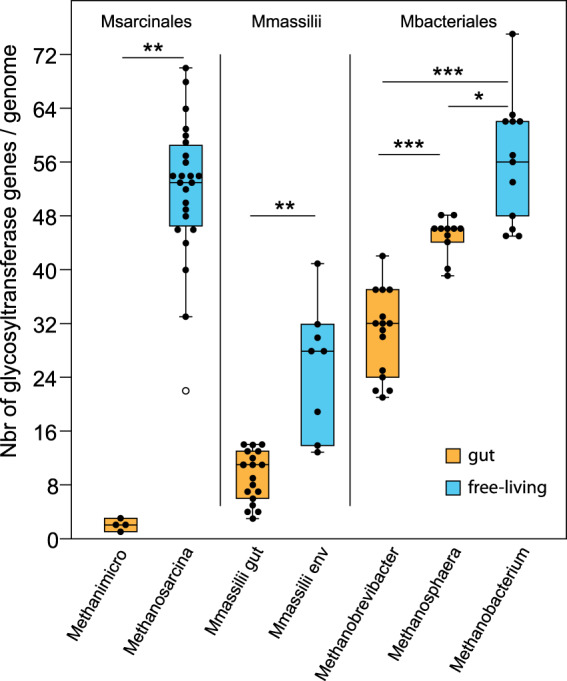
Number of glycosyltransferase genes in host-associated and environmental methanogens belonging to Methanosarcinales (*n* = 28), Methanomassiliicoccales (*n* = 26) and Methanobacteriales (*n* = 38). For Methanosarcinales and Methanobacteriales, number of glycosyltransferases genes was compared between host-associated genera and the closest related genus (i.e., *Methanosarcina* for *Methanimicrococcus*; *Methanobacterium* for *Methanobrevibacter* and *Methanosphaera*). The three *Methanimicrococcus* MAGs were added to this analysis. For Methanomassiliicoccales, “*Ca*. M. intestinalis” and *Methanomethylophilaceae* MAGs were pooled in “Mmassilii gut” and all other MAGs in “Mmassilii env”. Stars represent the significant differences between groups, determined using a Wilcoxon test (continuity correction: ***p.adj < 1e−4, **p.adj < 0.001, *p.adj < 0.05). Methanimicro *Methanimicrococcus*, Msarcinales Methanosarcinales, Mmassilii Methanomassiliicoccales, Mbacteriales Methanobacteriales.

While *M. blatticola* PA has kept the potential to express major S-layer proteins present in other Methanosarcinales, its cell surface was likely highly modified through the loss of many enzymes involved in the synthesis of nucleotide-activated sugars that are precursors of cell-surface glycoconjugates/extracellular polysaccharides (Figs. 1c and 2). Moreover, *M. blatticola* PA has lost up to 35 glycosyl transferases (GT), including 12 with transmembrane domains. Within Archaea, this is the genome that currently encodes the lowest number of GTs (2) (Table S2) and it also contrasts with other Methanosarcinales that have 33 GTs genes on average (Table S5). Even if not to the same extent, other host-associated methanogens (Methanobacteriales and Methanomassiliicoccales) also tend to have a lower number of GTs than their closest free-living relatives (Fig. 3), suggesting that the loss of GTs is another convergent characteristic related to the adaptation to gut conditions in archaea. The two GTs conserved in *M. blatticola* PA are involved in N-glycosylation of cell surface proteins by (i) transferring the first nucleotide-activated sugar on the lipid carrier (AglJ) and (ii) transferring the (oligo)saccharide from the lipid carrier to the nascent protein (AglB). Considering the almost complete loss of GTs and nucleotide-activated sugars synthesis genes, the nature of the glycans on the N-glycosylated proteins remains unclear (Fig. 2). Four membrane-bound proteins of unknown function and one with Flg_new domains have a PGF-CTERM domain, indicating that they are recognized by the archeosortase A present in *M. blatticola* PA and possibly N-glycosylated [44]. The number of such proteins is much lower in *M. blatticola* PA compared to other Methanosarcinales, which on average encode 20 proteins with this signal, suggesting a lower number of N-glycosylated proteins in *M. blatticola* PA (Table S5). Moreover, *M. blatticola* PA has lost two archaeosortases systems (PEF-CTERM and VPXXXP-CTERM) that may be involved in export and posttranslational modification of additional membrane-bound proteins [44].

Glycans originating from commensal microorganisms cover many roles in the digestive tract, including persistence in this environment, microbe–microbe interactions, protection from phages, and host–microbe interactions, such as host-immune system stimulation [45]. However, there are no data on the role of archaeal glycans in the host microbiome and very few on the cell wall of the *Methanosarcinaceae* members outside the *Methanosarcina* genus. When they are aggregated, *Methanosarcina* members are surrounded by methanochondroitin [46], a fibrillar polymer that is analogous to the chondroitin present in tissues of animals, including cockroaches [47]. The ability to synthesize two of the sugars that constitute methanochondroitin (*N*-acetylgalactosamine and glucuronic acid) was lost in *M. blatticola* PA (Fig. 2). To our knowledge, such a large loss of genes associated with cell-surface glycoconjugates or exopolysaccharide synthesis has never been reported in the archaea, and how this event is linked to adaptation of *M. blatticola* to the digestive tract remains to be elucidated.

### Adaptations to the gut as a nutrient/substrate rich environment

Several other gene losses reflect an adaptation to the gut as a nutrient/substrate-rich environment (Table 1), relative to non-gut environments that are essentially oligotrophic [48]. *M. blatticola* PA has lost a high-affinity phosphate transporter (*pstABC*) and an alkaline phosphatase (*phoA*) for organo-phosphate utilization, both of which are known to be upregulated under low phosphate concentrations in *Methanosarcina mazei* [49]. In contrast, the PitA low-affinity phosphate transporter [50] was kept in *M. blatticola* PA. Moreover, *M. blatticola* PA has lost the capacity to fix atmospheric nitrogen (nitrogenase *nifKDU* and regulatory genes P-II) and carbon dioxide (*cdhAB*) for autotrophic growth. The loss of these genes has also been previously reported in other gut methanogens belonging to *Methanosphaera* and *Methanobrevibacter* [51], and a loss of the nitrogenase was also suggested in host-associated Methanomassiliicoccales [52]. In contrast, termite-associated *Methanobrevibacter* spp. have not lost these genes [51], which may reflect adaptation to a different gut compartment or a lesser degree of specialization to the gut environment. The dependence of *M. blatticola* PA to a nutrient-rich environment is also reflected by the loss of several genes involved in amino acid synthesis (e.g., *asnB* for asparagine, *trpABDFGE* for tryptophan). The gene *cstA* was acquired in *M. blatticola* PA, possibly allowing the uptake of organic carbon and nitrogen in the form of pyruvate and peptides, as reported in gut-associated bacteria [53, 54]. This gene was independently acquired in a gut-associated Methanomassiliicoccales (*Ca.* Methanomassiliicoccus intestinalis Mx-01) suggesting convergent adaptation (Table 1 and Fig. S3). All these characteristics fit well with the high growth requirements of *M. blatticola* PA (acetate, yeast extract, tryptic soy, and vitamins [11]), as compared to most other *Methanosarcinaceae* members [42]. With the loss of *comDE* genes coding for coenzyme-M, the dependency of *M. blatticola* PA on other members of the gut microbiota includes this key cofactor of methanogenesis, consistently with the previously reported need of *M. blatticola* PA for an external source of this coenzyme [11]. The absence of *comDE* genes has been previously reported in several other gut methanogens such as *Methanobrevibacter ruminantium* [8] and several Methanomassiliicoccales (ISO4-H4 [55], ISO4-G1 [56]), including the termite-associated *Methanoplasma termitum* [9].

Seven markers recently identified as co-occurring with MCR and that are mostly conserved in Class I/Class II methanogens [18] were lost in *M. blatticola* PA. Three of them (m19, m21, and m24) are also missing in methanogens from nutrient-rich environments, including the host-associated Methanomassiliicoccales (m19 and m21) and host-associated Methanobacteriales (m21 and m24) (Tables 1 and S6; [18]). The function of these genes is currently unknown, but they have been suggested to be involved in regulatory processes of methanogenesis related to changes in substrate/nutrient availability [18]. Various methanogens, including *Methanosarcinaceae* [57, 58], are able to store carbon/energy as glycogen when substrates are available and to use it in periods of starvation. Genes involved in glycogen synthesis and degradation and those involved in glycolysis were lost in *M. blatticola* PA (Tables 1 and S4), indicating an adaptation to more stable conditions in terms of substrate availability.

The lower variability in the conditions faced by *M. blatticola* PA are also reflected in a drastically reduced capacity to sense its environment and move to more favorable conditions (Fig. 1C), with the loss of the *cheABCFDRWY* chemotaxis genes, 19 histidine kinase/PAS sensor genes, and the whole motility machinery (*flaBCEFGH*) (Tables 1 and S4). In addition, 43 genes coding for transcriptional regulators were lost. This matches previous observations reported for gut-associated *Methanobrevibacter*/*Methanosphaera* and Methanomassiliicoccales, which have a significantly smaller number of genes involved in chemotaxis, signal transduction, and transcriptional regulation, as compared to non-gut methanogens [6, 52] (Table 1). These characteristics are also consistent with what has been reported from gut bacteria, which have generally a smaller repertoire of genes involved in motility, chemotaxis [59], and transcriptional regulation [60], relative to non-gut bacteria (Table 1). These results reveal the existence of largely shared traits of adaptation to a host-associated lifestyle in both archaea and bacteria.

### A unique case of loss of the H_4_MPT methyl-branch of the Wood–Ljungdahl pathway among Class I/II methanogens and evolution toward methyl-reducing hydrogenotrophy

**Fig. 4:**
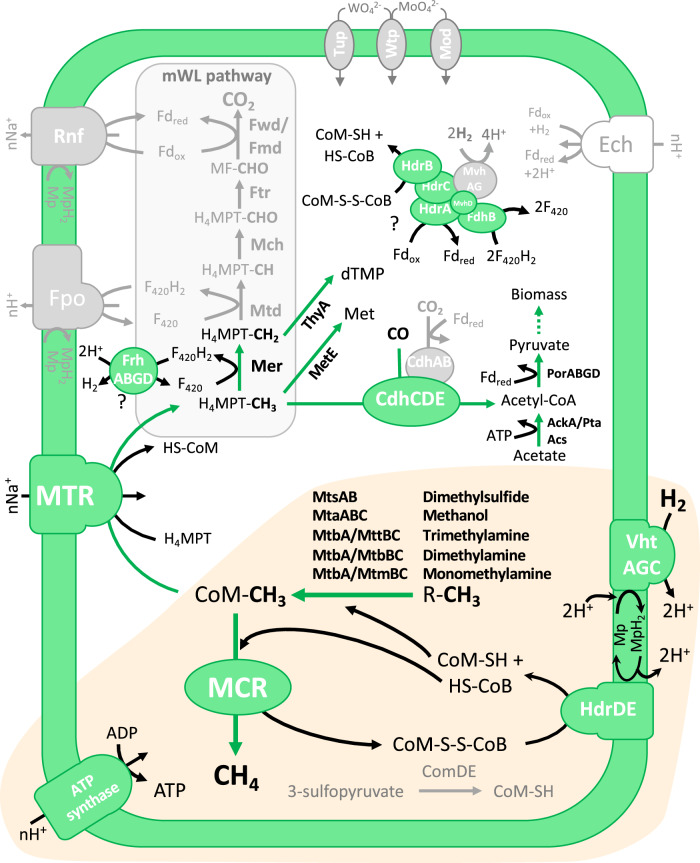
Methanogenesis-associated enzymes in *M. blatticola* PA: losses and role in catabolism and anabolism. Enzymes in gray were lost in *M. blatticola* PA. The Ech complex (in white) was not present in the last common ancestor of *Methanimicrococcus* and *Methanosarcina* but likely acquired in *Methanosarcina*. Enzymes/enzymatic complexes within the beige frame (MCR, VhtAGC, HdrDE, MtsAB, MtaABC, MtbA, MttBC, MtbBC, MtmBC, and ATP synthase) are involved in energy conservation. Other enzymes/enzymatic complexes that can be involved in methane metabolisms in other archaea but predicted to be involved in anabolism in *M. blatticola* PA: MTR complex, Frh, Mer, AckA/Pta, Acs, CdhCDE, and the putative HdrABC/MvhD/FdhAB-like complex. CdhCDE may also have a role in CO detoxification. The full names of the proteins are displayed in Table S4.

Among the enzymes needed for energy conservation, the most notable losses correspond to several genes involved in methanogenesis, particularly those coding for the H_4_MPT methyl branch of the Wood–Ljungdahl (H_4_MPT mWL) pathway (*fwdABCDEFG*, *fmdBD*, *ftr*, *mch*, and *mtd*; Fig. 4 and Table 1). Moreover, these losses also include the genes coding for the biosynthesis of two cofactors of Fwd/Fmd, (methanofuran (*mfnBDEF*) and molybdopterin (*moaABC*/*mobAB*/*moeA*)) as well as those used to import the ions associated with these complexes (molybdate (*modABCDE*, *wtpAB*) and tungstate (*tupAB*, *wtpAB*)). Altogether, this explains the inability of *M. blatticola* PA to grow by reducing CO_2_ with H_2_ or by the disproportionation of methyl compounds (methylotrophic methanogenesis) as reported by Sprenger et al. [11]. This pathway was previously suggested to be absent in *M. blatticola* PA [16], but it was only inferred indirectly by the low enzymatic activity of the F_420_-reducing hydrogenase, which did not provide information on the presence/absence of the genes of the H_4_MPT mWL pathway. The almost complete absence of the genes of the H_4_MPT mWL pathway is remarkable, as it has never been previously observed in a member of Class I (Methanobacteriales, Methanopyrales, Methanococcales) and Class II (Methanosarcinales, Methanomicrobiales, Methanocellales) methanogens, even in those that do not use it for methanogenesis, such as *Methanosphaera stadtmanae* [61].

Interestingly, one of the genes of the H_4_MPT mWL pathway (*mer*) and those coding for the MTR complex were kept (Fig. 4). In the absence of the remaining components of the H_4_MPT mWL pathway, their implication in methanogenesis is unlikely. However, these enzymes are presumably functional, as *M. blatticola* PA has also retained the capacity to synthesize their associated cofactors, H_4_MPT (Table S4; [62]) and F_420_ (Fig. 4). The presence of the F_420_ cofactor is supported by the previously reported observation of fluorescence at 420-nm excitation in *M. blatticola* PA [11]. Mer and MTR may have been kept in *M. blatticola* PA for their anabolic role, as suggested by the presence of MetE and ThyA homologs, possibly involved in the synthesis of methionine using H_4_MPT-CH_3_ [63] and thymidylate (dTMP) using H_4_MPT-CH_2_ [64] (Fig. 4). Consistently with this hypothesis, it was shown that *M. barkeri* growing on methanol and H_2_ is dependent on Na^+^ for growth, but not for ATP and methane formation [65]. This further supports the potential anabolic role of MTR (that is Na^+^ dependent) during the growth of *Methanosarcinaceae* spp. on methyl compounds with H_2_. In *M. blatticola* PA, the methyl and methylene groups needed for these anabolic reactions would thus be mainly provided by the methyl compounds used for methanogenesis (Fig. 4). The methylene groups may additionally be derived from formaldehyde produced by the pentose phosphate pathway [66], because a bifunctional Fae-Hps enzyme (a fused formaldehyde-activating/3-hexulose-6-phosphate synthase) is encoded by *M. blatticola* PA.

The specialization of *M. blatticola* PA on methyl-reducing hydrogenotrophic methanogenesis was also likely associated with loss of the membrane-bound Fpo and Rnf complexes (Fig. 4), which are involved in energy conservation in other Methanosarcinales [67]. The absence of the Fpo complex and of an Ech complex in *M. blatticola* PA was previously reported [68]. The Ech complex, which was likely acquired in *Methanosarcina*, is notably used to reduce ferredoxin for anabolic purpose in species growing by reduction of methanol with H_2_ [69]. One can therefore wonder how *M. blatticola* PA reduces ferredoxin for anabolic purposes (e.g., pyruvate synthesis; Fig. 4). An HdrABC/MvhD/Fd(r)hB complex may play this role by reducing ferredoxin and CoM-S-S-CoB using F_420_H_2_ (generated during the oxidation of H_4_MPT-CH_3_ by Mer, or by FrhABG using H_2_ as electron donor) through electron bifurcation, in a similar way as the HdrABC/MvhADG complex [70] but using F_420_H_2_ instead of H_2_ (Fig. 4). This potential complex was first reported in a methanotrophic member of the Methanosarcinales (ANME-2d) where it was referred as HdrABC/MvhD/FrhB and proposed to perform electron confurcation (the reverse reaction of what we propose to occur in *M. blatticola*) for ferredoxin and CoM-S-S-CoB recycling [71]. This putative complex is encoded by a gene cluster that was reported in many methane and short-chain alkane-oxidizing archaea [18].

Except for *M. blatticola* PA, all other *Methanosarcinaceae* representatives have the capacity to disproportionate methyl compounds. Thus, the specialization of *M. blatticola* PA on methyl-reducing hydrogenotrophic methanogenesis likely emerged from this type of metabolism. For methyl-compound disproportionation, *Methanosarcinaceae* spp. use the H_4_MPT mWL pathway to oxidize one methyl group into CO_2_, producing three reducing equivalents for the reduction of three additional methyl groups into CH_4_ [72]. However, when *M. barkeri* grows on methanol in the presence of H_2_, methanol is totally reduced by H_2_ and no longer oxidized into CO_2_ [65], suggesting that the H_4_MPT mWL pathway is no longer used for methanogenesis under such conditions. It is thus possible that an ancestor of *M. blatticola* PA lost the genes encoding the H_4_MPT mWL pathway after stable colonization of the animal gut environment where both H_2_ and methyl compounds are available. Indeed, the concentrations of H_2_ in the gut (4 μM in cockroaches [17]; 100 nM–50 μM in ruminants [73]; 168 μM in mice [74]; 5–156 μM in humans [75, 76]) are generally 10–1000-folds higher than those in anoxic soils or sediments (10–30 nM [77, 78]). This is likely due to the constant load of fresh organic matter making H_2_ production rates much higher in the animal gut (5 μM h^−1^ and 2 mM h^−1^ [79]) than in the environment (1 μM h^−1^ in eutrophic lake sediments [77]). For similar reasons, methyl-compound concentrations in the gut (e.g., for methanol, 10 μM in cockroaches [17]; 23–72 μM in the rumen [80]; 70 μM in humans [3]) are also higher than those in sediments, where they are generally around or below the micromolar level [81, 82]. The availability of methanol and hydrogen also potentially drove the specialization on methyl-reducing hydrogenotrophic methanogenesis in the members of the genus *Methanosphaera* (Class I methanogen, Methanobacteriales). Several Methanobacteriales have the *mtaABC* genes for methanol utilization, but except *Methanosphaera* spp. only a few have been shown to grow on methanol + H_2_ [83, 84]. It is therefore possible that the ancestors of *Methanosphaera* spp. evolved stepwise from (i) CO_2_-reducing methanogenesis (which is shared by all Methanobacteriales) to (ii) facultative methyl-reducing methanogenesis and then to (iii) obligate methyl-reducing methanogenesis. The transition from facultative to obligate methyl-reducing methanogenesis likely occurred when molybdopterin biosynthesis genes (absent in *Methanosphaera spp*. [51, 61] were lost. All the main enzymes of the H_4_MPT mWL pathway are present in *Methanosphaera* and involved in anabolic reactions [61].

With *M. blatticola* PA, the only other known methanogens missing part or all of the H_4_MPT mWL pathway belong to recently discovered lineages branching all over the tree of archaea: Methanomassiliicoccales [22] Methanofastidiosa [19], Verstraetearchaeota [21], Methanonatronarchaeia [20], and NM3 Acherontia [18]. All these methanogens have been experimentally characterized or predicted to be methyl-reducing hydrogenotrophic methanogens. The origin of this metabolism in these evolutionarily distant lineages has been discussed and investigated previously [18, 85]. Briefly, methyl-reducing hydrogenotrophic methanogenesis could have originated early in the evolution of archaea and been vertically inherited in these lineages or rather have been acquired by horizontal gene transfers and/or emerged through the loss of the H_4_MPT mWL pathway and the MTR complex. These hypotheses are not mutually exclusive, and according to phylogenetic analyses of the MCR complex, the acquisition of this metabolism via horizontal gene transfer is very likely at least for some of these lineages [18]. The hypothesis of the emergence of methyl-reducing hydrogenotrophic methanogenesis from methanogenesis involving the H_4_MPT mWL pathway is difficult to test in these deep-branching lineages. The case of *M. blatticola* therefore represents the first concrete proof of a partial loss of the H_4_MPT mWL pathway in a methanogen. When methanogens stop using the H_4_MPT mWL pathway for methanogenesis, they tend to keep these enzymes for anabolic purposes, but whether those enzymes are replaced by other enzymes over long evolutionary periods remains unknown.

### Three novel MAGs support the patterns of gene gains/losses in *Methanimicrococcus*

During the course of this analysis, three *Methanimicrococcus* MAGs from a termite gut sample were released in GenBank [86] (Table S3 and Fig. S4). By comparing the protein sequences of these MAGs with those of *M. blatticola* PA, we observe that most of the genes (93.3%) that we predicted to have been lost in *M. blatticola* PA are also absent in these three MAGs (Table S4). This shows that most of these losses occurred before the LCA of these four *Methanimicrococcus* species. The proteins absent in the three *Methanimicrococcus* MAGs cover those discussed above and are involved in methanogenesis (mWL pathway and MCR-associated markers), glycogen synthesis/utilization, chemotaxis, nucleotide-activated sugar synthesis, GTs (1–3 only are present, Fig. 3), and 86% (37) of the transcriptional regulators lost in *M. blatticola* PA (Table 1). Only 0.01% of genes predicted to be lost in *M. blatticola* PA are present in all 3 MAGs and could therefore represent losses specific to this species. A smaller fraction of the genes that were gained in *M. blatticola* PA are also present in the 3 *Methanimicrococcus* MAGs: 50% are present in at least 1 of them and only 19.4% are present in all 3 MAGs, suggesting that there is a weaker pattern of gene acquisition than gene loss. However, some of the gained proteins—in particular ALPs—may have evolved faster than average following acquisition and could have been missed by our identity threshold. For example, no genes coding for homologs of the *M. blatticola* PA proteins with a Flg_new domain were found in the 3 MAGs using a 40% identity threshold, but we detected 3 (in the least complete MAG) to 13 proteins with a Flg_new domain in the proteome of these MAGs using a specific HMM search.

## Concluding remarks

Our results uncover how the transition from the open environment to the gut can deeply modify the physiology of an archaeon, including its central energy metabolism. Moreover, it reveals one of the possible paths toward the emergence of methyl-reducing hydrogenotrophic methanogenesis. In contrast to Methanomassiliicoccales, *M. blatticola* PA and *Methanosphaera* spp. have specialized relatively recently (at the genus level) on methyl-reducing hydrogenotrophic methanogenesis. This probably occurred after gut colonization, being triggered by the conditions encountered in this environment. After they became obligate methyl-reducing hydrogenotrophic methanogens, *M. blatticola* PA and *Methanosphaera* spp. kept one or all of the H_4_MPT mWL enzymes, respectively, for the synthesis of amino acids and purines. This may represent an intermediate state toward the complete loss of H_4_MPT mWL pathway and the MTR complex, as observed in the deep-branching lineages of obligate methyl-reducing hydrogenotrophic methanogens that were recently discovered [18–22]. There are multiple other (non-exclusive) explanations for the presence of this metabolism in free-living methanogens [18]: (i) it could have arisen from horizontal gene transfer to previously non-methanogenic archaea, (ii) the mWL pathway could have been lost in CO_2_-reducing methanogens thriving in some specific non-gut environment where the conditions are favorable to this type of methanogenesis, and (iii) the methyl-reducing hydrogenotrophic methanogenic pathway may be the ancestral type of methanogenesis. In this latter case, the specialization of *Methanimicrococcus* and *Methanosphaera* on this type of metabolism because of gut colonization would correspond to a step back to the original type of methanogenesis.

Our analysis also highlights multiple adaptations to the digestive tract of animals, several of which have also been previously proposed in phylogenetically distant host-associated archaea belonging to the Methanobacteriales and Methanomassiliicoccales. These convergent adaptations include the loss of the ability to grow autotrophically, to fix nitrogen, and of specific methanogenesis/MCR-associated markers. They also indicate a reduced capacity to sense and respond to variations in the environment, and an important shift in cell surface elements, notably by reduction in the number of GTs and gain of ALPs such as the Listeria-Bacteroidetes repeats/Flg_New domain proteins. The gain and loss of some of these genes also fit patterns of gene depletion or enrichment observed in gut bacteria, revealing common adaptation mechanisms across the two prokaryotic domains. Several gene losses observed in *M. blatticola* PA, and suggested in other gut methanogens, are also similar to those reported for two methanogens endosymbionts of ciliates, *Methanobrevibacter* sp. NOE and *Methanocorpusculum* sp. MCE, affiliated to Methanobacteriales and Methanomicrobiales, respectively [87]. Indeed, these two endosymbionts have also lost transcriptional regulators, aromatic amino acid biosynthesis, and cell surface modification enzymes [87]. Beyond the gut environment, some of these losses could thus be more generally indicative of host adaptation.

Currently, the availability of archaeal genomes associated with a host is essentially restricted to humans, ruminants, and, to a lesser extent, termites and sponges. Sequencing of new archaeal genomes from a wide variety of animal hosts will therefore provide key information to draw a comprehensive picture of host-adaptation mechanisms in the archaea.

## Supplementary information

Supplementary Figure 1.

Supplementary Figure 2.

Supplementary Figure 3.

Supplementary Figure 4.

Supplementary Table 1.

Supplementary Table 2.

Supplementary Table 3.

Supplementary Table 4.

Supplementary Table 5.

Supplementary Table 6.

Supplementary Data Set 1.

Supplementary Data Set 2.

Supplementary Data Set 3.

Supplementary Data Set 4.
