# Supplementary material for: Comparative genomic analysis of Methanimicrococcus blatticola provides insights into host adaptation in archaea and the evolution of methanogenesis
Source: ISME Commun. 2021 Sep 9;1:47. doi: 10.1038/s43705-021-00050-y (PMC9723798; doi:10.1038/s43705-021-00050-y)
Supplement: Supplementary file 1 — Supplementary Figure 1. [file 43705_2021_50_MOESM1_ESM.pdf]

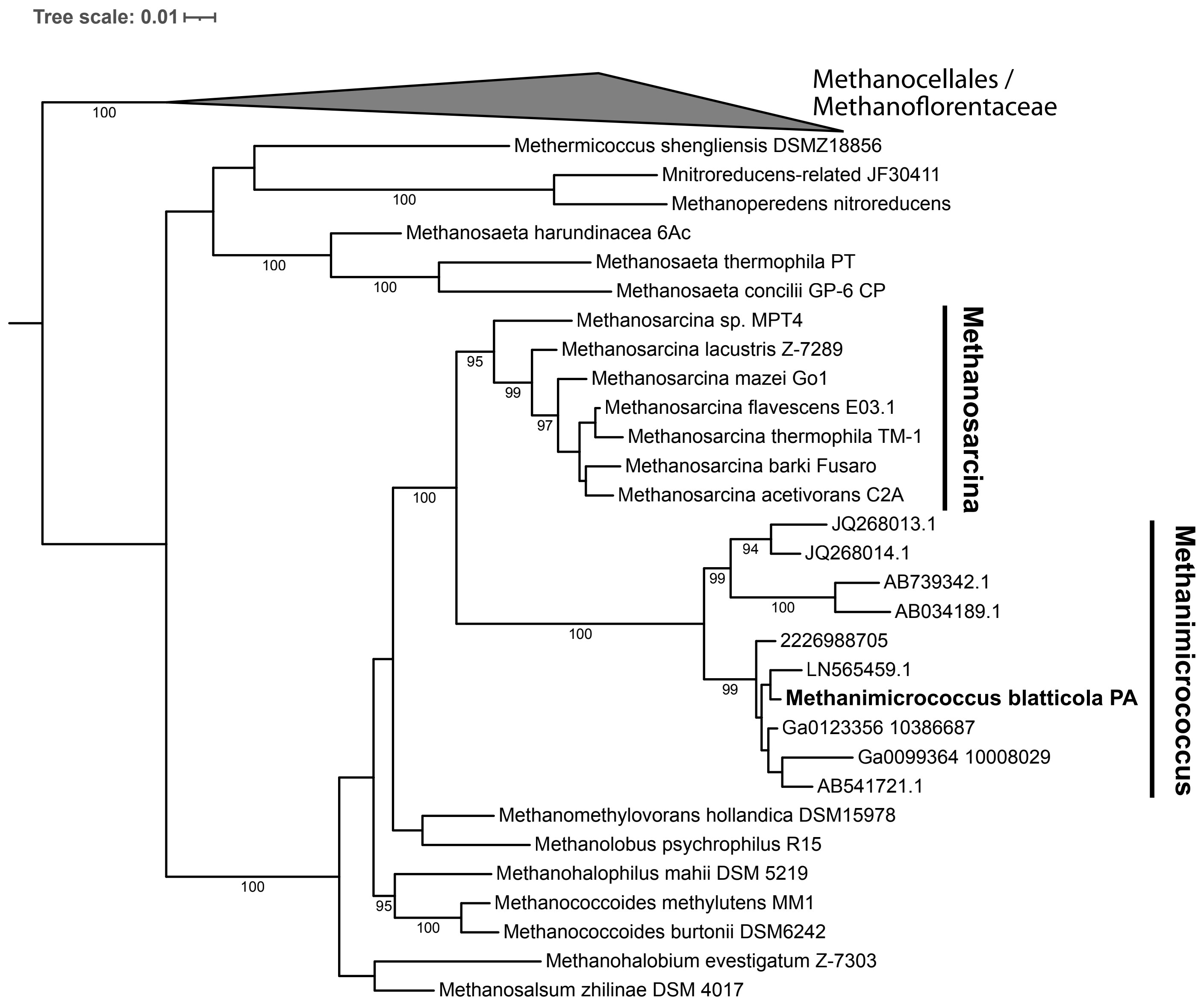

Figure S1: Phylogenetic position of Methanimicrococcus blatticola PA using 16S rRNA gene sequences (1,431 positions; Maximum likelihood inference with GTR+R3). Branch support (ultrafast bootstraps; 1000 iterations) above 90 are shown.
