# Supplementary material for: Comparative genomic analysis of Methanimicrococcus blatticola provides insights into host adaptation in archaea and the evolution of methanogenesis
Source: ISME Commun. 2021 Sep 9;1:47. doi: 10.1038/s43705-021-00050-y (PMC9723798; doi:10.1038/s43705-021-00050-y)
Supplement: Supplementary file 2 — Supplementary Figure 2. [file 43705_2021_50_MOESM2_ESM.pdf]

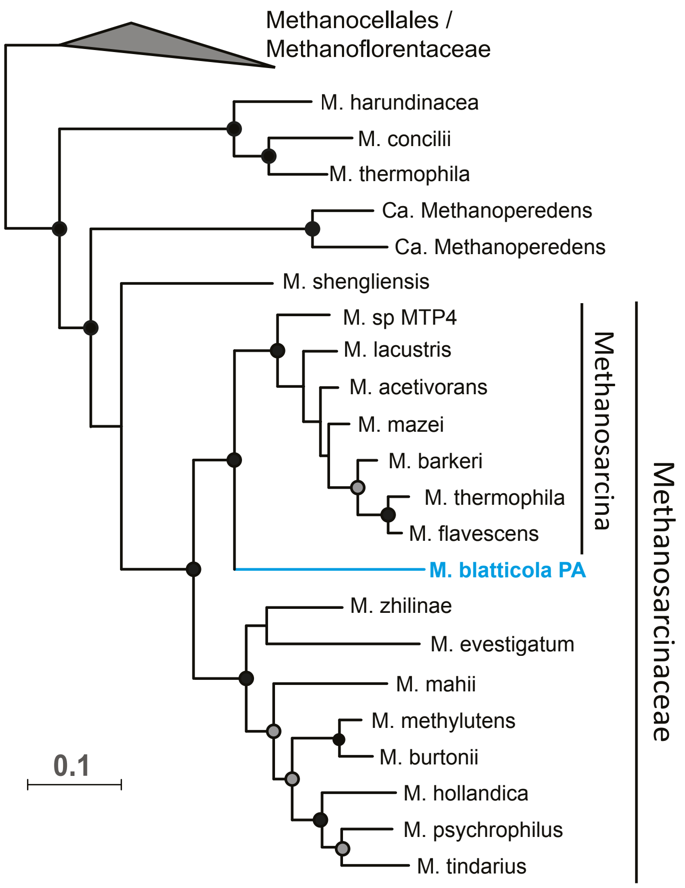

Figure S2: Maximum likelihood phylogeny (IQ-TREE, LG+F+Γ4) of the Methanosarcinales based on a concatenation of McrABG (1,176 positions). The color of the circles at the nodes indicates ultrafastbootstrap values (black, >95; grey, between 70 and 95).
