# Supplementary material for: Comparative genomic analysis of Methanimicrococcus blatticola provides insights into host adaptation in archaea and the evolution of methanogenesis
Source: ISME Commun. 2021 Sep 9;1:47. doi: 10.1038/s43705-021-00050-y (PMC9723798; doi:10.1038/s43705-021-00050-y)
Supplement: Supplementary file 3 — Supplementary Figure 3. [file 43705_2021_50_MOESM3_ESM.pdf]

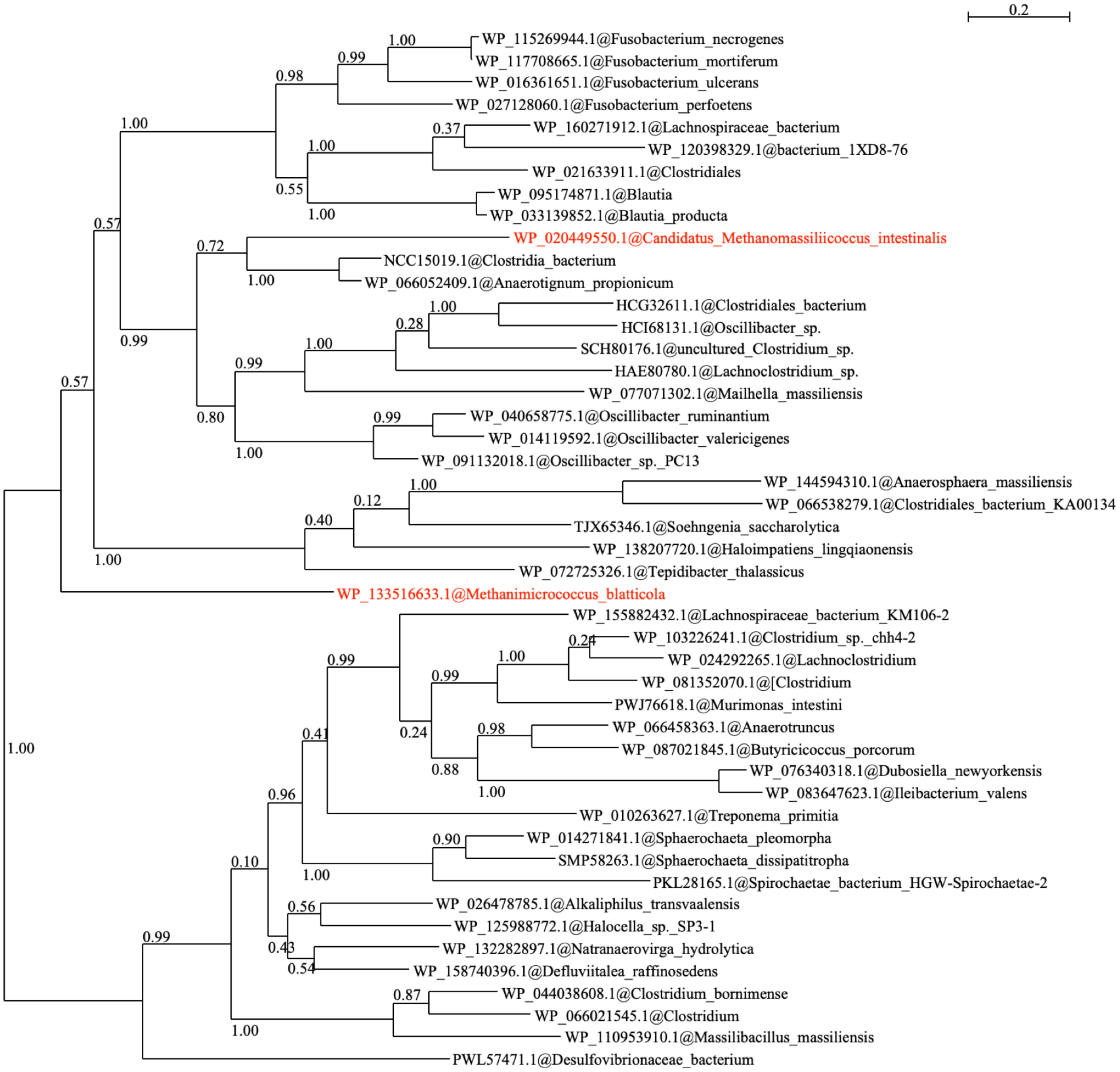

Figure S3: Maximum likelihood (LG+G4) tree of CstA (668 positions). Sequences of methanogens are in red, those of bacteria are in black.
