# Supplementary material for: Comparative genomic analysis of Methanimicrococcus blatticola provides insights into host adaptation in archaea and the evolution of methanogenesis
Source: ISME Commun. 2021 Sep 9;1:47. doi: 10.1038/s43705-021-00050-y (PMC9723798; doi:10.1038/s43705-021-00050-y)
Supplement: Supplementary file 5 — Supplementary Table 1. [file 43705_2021_50_MOESM5_ESM.pdf]

Table S1: Genome characteristics of *M. blatticola* PA and comparison with genomes used for the analysis of gene gains and losses. Colors range from blue for the lowest values to orange for the highest ones.

NCBI TaxID	Species name	Order	Family	Contig number	Size (bp)	Protein nbr	GC%	Genome/ MAG	Isolation source[a]
**91560**	**Methanimicrococcus blatticola PA**	**Methanosarcinales**	**Methanosarcinaceae**	**12**	**1783575**	**1569**	**42**	**Genome**	**Periplaneta americana hindgut**
188937	Methanosarcina acetivorans C2A	Methanosarcinales	Methanosarcinaceae	1[b]	5751492	4567	42,7	Genome	Marine sediment
269797	Methanosarcina barkeri Fusaro	Methanosarcinales	Methanosarcinaceae	1[b]	4873766	3739	39,3	Genome	Lake sediment
1715806	Methanosarcina flavescens	Methanosarcinales	Methanosarcinaceae	74	3283688	2846	41,31	Genome	Biogas plant
1434111	Methanosarcina lacustris Z-7289	Methanosarcinales	Methanosarcinaceae	1[b]	4139808	3264	41,8	Genome	Lake sediment
192952	Methanosarcina mazei Go1	Methanosarcinales	Methanosarcinaceae	1[b]	4096345	3347	41,5	Genome	Sewage digester
1434100	Methanosarcina sp. MTP4	Methanosarcinales	Methanosarcinaceae	1[b]	4211468	3422	45,9	Genome	Marine sediment
523844	Methanosarcina thermophila TM-1	Methanosarcinales	Methanosarcinaceae	1[b]	3127379	2597	41,1	Genome	Sludge digestor
259564	Methanococcoides burtonii DSM 6242	Methanosarcinales	Methanosarcinaceae	1[b]	2575032	2406	40,8	Genome	Lake hypolimnion
1434104	Methanococcoides methylutens MM1	Methanosarcinales	Methanosarcinaceae	1[b]	2394636	2245	44	Genome	Marine sediment
644295	Methanohalobium evestigatum Z-7303	Methanosarcinales	Methanosarcinaceae	1[b]	2406232	2157	36,6	Genome	Saline lagoon
547558	Methanohalophilus mahii DSM 5219	Methanosarcinales	Methanosarcinaceae	1[b]	2012424	1955	42,6	Genome	Lake sediment
1094980	Methanolobus psychrophilus R15	Methanosarcinales	Methanosarcinaceae	1[b]	3072769	2841	44,6	Genome	Wetland
1090322	Methanolobus tindarius DSM 2278	Methanosarcinales	Methanosarcinaceae	1[b]	3151883	2917	39,8	Genome	Marine sediment
867904	Methanomethylovorans hollandica DSM 15978	Methanosarcinales	Methanosarcinaceae	1[b]	2714013	2281	42,6	Genome	Freswater pond sediment
679901	Methanosalsum zhilinae DSM 4017	Methanosarcinales	Methanosarcinaceae	1[b]	2138444	1955	39,2	Genome	Alkaline lake sediment
1392998	Candidatus Methanoperedens nitroreducens	Methanosarcinales	Ca. Methanoperedenaceae	72 (10 scaffolds)	3203386	3435	43,1	MAG	Bioeactor
2035255	Candidatus Methanoperedens sp. BLZ2	Methanosarcinales	Ca. Methanoperedenaceae	85	3735583	3790	40,3	MAG	Bioeactor
990316	Methanosaeta concilii GP6	Methanosarcinales	Methanosaetaceae	1[b]	3026645	2878	51	Genome	Sewage sludge
1110509	Methanosaeta harundinacea 6Ac	Methanosarcinales	Methanosaetaceae	1[b]	2571034	2385	60	Genome	Sludge blanket reactor
349307	Methanosaeta thermophila PT	Methanosarcinales	Methanosaetaceae	1[b]	1879471	1696	53,5	Genome	Thermophilic digester
1122233	Methermicoccus shengliensis DSM 18856	Methanosarcinales	Methermicoccaceae	15	1513583	1611	54,7	Genome	Offshore oil field
351160	Methanocella arvoryzae MRE50	Methanocellales	Methanocellaceae	1[b]	3179916	3058	54,6	Genome	Rice field
1041930	Methanocella conradii HZ254	Methanocellales	Methanocellaceae	1[b]	2378438	2436	52,7	Genome	Rice field
304371	Methanocella paludicola SANAE	Methanocellales	Methanocellaceae	1[b]	2957635	3004	54,9	Genome	Rice field
Na[c]	Methanoflorens stordalenmirensis	Methanoflorentales	Methanoflorentaceae	117 (10 scaffolds)	2064259	2373	51,58	MAG	Thawing permafrost

Information about *Methanimicrococcus blatticola* PA sequenced in this study are in bold. [a]Origin of the strain/enrichment culture/sample from which the genome/MAG was sequenced, [b]closed genome, [c]this MAG is available in IMG database (ID 2518645542)
