# Supplementary material for: Comparative genomic analysis of Methanimicrococcus blatticola provides insights into host adaptation in archaea and the evolution of methanogenesis
Source: ISME Commun. 2021 Sep 9;1:47. doi: 10.1038/s43705-021-00050-y (PMC9723798; doi:10.1038/s43705-021-00050-y)
Supplement: Supplementary file 7 — Supplementary Table 3. [file 43705_2021_50_MOESM7_ESM.pdf]

Table S3: Characteristics of three recently released *Methanimicrococcus* MAGs.

NCBI TaxID	Assemby	Contig number	Size (bp)	Protein number	GC%	Comp (%)	Conta (%)	Isolation source[a]
2022468	GCA009784005	62	1688578	1532	45.5	92.14	0.00	Labiotermes labralis P3 gut compartment
2022468	GCA009783635	138	1333240	1316	45.9	84.52	2.29	Labiotermes labralis P3 gut compartment
2022468	GCA009776675	239	1397194	1511	39.6	75.09	4.58	Termes hospes gut

Comp, Completion; Conta, Contamination
