# Supplementary material for: Comparative genomic analysis of Methanimicrococcus blatticola provides insights into host adaptation in archaea and the evolution of methanogenesis
Source: ISME Commun. 2021 Sep 9;1:47. doi: 10.1038/s43705-021-00050-y (PMC9723798; doi:10.1038/s43705-021-00050-y)
Supplement: Supplementary file 9 — Supplementary Table 5. [file 43705_2021_50_MOESM9_ESM.pdf]

Table S5: Number of proteins with a PGF-CTERM signal (likely N-glycosylated) and number of glycosyltransferase families in the Methanosarcinales members. The PGF-CTERM signal was first identified using TIGR04126 hmm profile, and manually refined profile targeting more specifically the Methanosarcinales sequences. Glycosyltransferases families were retrieved from CAZy database (http://www.cazy.org/), with the exception of M. *blatticola* PA, *Methanolobus tindarius* DSM 2278, *Methermicoccus shengliensis* DSM 18856 and the two '*Ca*. Methanoperedens' MAGs, that were annotated using dbCAN2. Colors range from blue for the lowest values to orange for the highest ones.

Species	Taxon family	PGF-CTERM proteins	Glycosyltransferases families												Total GTs
			1	2	3	4	20	35	66	81	83	84	94	NC	
**Methanimicrococcus blatticola PA**	**Methanosarcinaceae**	**5**	**0**	**0**	**0**	**0**	**0**	**0**	**1**	**1**	**0**	**0**	**0**	**0**	**2**
Methanosarcina acetivorans C2A	Methanosarcinaceae	44	3	13	1	20	0	1	4	0	0	0	0	0	42
Methanosarcina barkeri Fusaro	Methanosarcinaceae	34	2	21	1	23	0	2	3	0	0	0	0	0	52
Methanosarcina flavescens	Methanosarcinaceae	30	3	17	1	20	0	1	3	0	0	0	0	0	45
Methanosarcina lacustris Z-7289	Methanosarcinaceae	19	2	20	1	31	0	1	2	0	0	0	0	0	57
Methanosarcina mazei Go1	Methanosarcinaceae	29	2	23	1	26	0	1	3	0	0	0	0	0	56
Methanosarcina sp. MTP4	Methanosarcinaceae	27	3	10	1	14	0	1	2	0	0	0	0	0	31
Methanosarcina thermophila TM-1	Methanosarcinaceae	13	1	18	1	21	0	1	3	0	0	0	0	0	45
Methanococcoides burtonii DSM 6242	Methanosarcinaceae	24	1	10	1	8	0	0	1	1	0	0	0	0	22
Methanococcoides methylutens MM1	Methanosarcinaceae	21	1	5	1	8	0	0	1	1	0	0	0	0	17
Methanohalobium evestigatum Z-7303	Methanosarcinaceae	9	1	5	0	8	0	1	1	1	0	0	0	0	17
Methanohalophilus mahii DSM 5219	Methanosarcinaceae	16	1	5	1	3	0	0	1	1	0	0	0	0	12
Methanolobus psychrophilus R15	Methanosarcinaceae	19	4	13	1	14	0	1	0	2	0	0	0	0	35
Methanolobus tindarius DSM 2278	Methanosarcinaceae	32	2	5	1	8	0	1	1	1	1	0	0	1	21
Methanomethylovorans hollandica DSM 15978	Methanosarcinaceae	6	0	7	1	18	0	1	2	0	0	0	0	0	29
Methanosalsum zhilinae DSM 4017	Methanosarcinaceae	18	0	2	1	3	0	1	1	2	0	0	0	1	11
Candidatus Methanoperedens nitroreducens	Methanoperedentaceae	26	0	23	0	17	1	0	2	0	6	0	0	1	50
Candidatus Methanoperedens sp. BLZ2	Methanoperedentaceae	25	0	27	0	12	1	0	2	0	2	0	1	2	47
Methanosaeta concilii GP6	Methanosaetaceae	2	1	16	1	15	0	2	2	0	0	1	0	1	39
Methanosaeta harundinacea 6Ac	Methanosaetaceae	1	0	8	1	9	0	4	4	0	0	0	0	0	26
Methanosaeta thermophila PT	Methanosaetaceae	2	0	7	1	10	1	1	3	0	0	0	0	1	24
Methermicoccus shengliensis DSM 18856	Methermicoccaceae	7	0	4	0	14	1	0	1	1	1	0	0	0	22

Glycosyltransferases families were retrieved from CAZy database (http://www.cazy.org/), with the exception of Methanimicrococcus blatticola, Methanolobus tindarius DSM 2278, Methermicoccus shengliensis DSM 18856 and the two Methanoperedens MAGs, that were annotated using dbCAN2.
