# Supplementary material for: Comparative genomic analysis of Methanimicrococcus blatticola provides insights into host adaptation in archaea and the evolution of methanogenesis
Source: ISME Commun. 2021 Sep 9;1:47. doi: 10.1038/s43705-021-00050-y (PMC9723798; doi:10.1038/s43705-021-00050-y)
Supplement: Supplementary file 10 — Supplementary Table 6. [file 43705_2021_50_MOESM10_ESM.pdf]

Table S6: Losses of "MCR-associated markers" in *Methanimicrococcus blatticola* PA and comparison with the prevalence of these genes in other gut-associated lineages and their phylogenetically closest non-gut relatives. "*Ca.* Methanomassiliicoccus intestinalis" (M. int.) is a Methanomassiliicoccaceae that is only present in the human gut. Mbac: Methanobacteriales; Mmass: Methanomassiliicoccales.

		**Gut-associated**	**Nbr of genomes / lineage**	m1_mcrA	m2_mcrB	m3_mcrG	m4_Predicted rotamase	m5_DUF2102	m6_DUF2112	m7_YjiL-like	m8_DUF2113	m9_arCOG03226	m10_atwA	m11_mcrC	m12_mcrD	m13_cfbD	m14_cfbE	m15_mtxX	m16_AIR-synthase-related	m17_Zn-ribbon protein	m18_DUF2099
**Methanosarcinaceae**	**Methanimicrococcus blatticola**	Y	1	MBLAb1417	MBLAb1421	MBLAb1418	MBLAb0808	MBLAb0807	MBLAb0806	MBLAb0805	MBLAb0804	MBLAb0803	MBLAb0809	MBLAb1419	MBLAb1420	MBLAb0389	MBLAb0388	MBLAb1464 MBLAb1101	MBLAb0230	MBLAb0116	MBLAb1068
	**Methanosarcina**	N	23	1	1	1	1	1	1	1	1	1	1	1	1	1	1	1	1	1	1
	**Other Methanosarcinaceae**	N	38	1	1	1	1	1	1	1	1	1	1	1	1	1	1	0.97	1	1	1
	**Methanosaetaceae**	N	3	1	1	1	1	1	1	1	1	1	1	1	1	1	1	1	1	1	1
**Mbac**	**Methanobrevibacter**	Y	17	1	1	1	1	1	1	1	1	1	1	1	1	1	1	1	1	1	0.94
	**Methanosphaera**	Y	5	1	1	1	1	1	1	1	1	1	1	1	1	1	1	1	1	1	1
	**Methanobacterium**	N	9	1	1	1	1	1	1	1	1	1	1	1	1	1	1	1	1	1	1
	**Methanothermobacter**	N	3	1	1	1	1	1	1	1	1	1	1	1	1	1	1	1	1	1	1
**Mmass**	**Methanomethylophilaceae**	Y	21	1	1	1	1	1	1	1	1	1	1	1	1	1	1	1	1	1	1
	**Methanomassiliicoccus intestinalis**	Y	5	1	1	1	1	1	1	1	1	1	1	1	1	1	1	1	1	1	1
	**Methanomassiliicoccaceae (no M. int.)**	N	6	1	1	1	1	1	1	1	1	1	1	1	1	0.5	0.5	1	1	1	0.83

		**Gut-associated**	**Nbr of genomes / lineage**	**m19_DUF2117**	m20_Thioglicine modif (McrA PTM)	**m21_DUF2124**	m22_DUF2098	m23_Soluble P-type ATPase	**m24_DUF2111**	m25_arCOG04853	m26_DUF2114	m27_mtrA	m28_mtrB	m29_mtrC	m30_mtrD	m31_mtrE	m32_arCOG04885	m33_Arg-methyltransf (McrA PTM)	m34_DUF1894	m35_DUF1890	m36_DUF2115	m37_DUF2119	m38_DUF2121
**Methanosarcinaceae**	**Methanimicrococcus blatticola**	Y	1	-	MBLAb0762	-	MBLAb1428	MBLAb0810	-	MBLAb1151	MBLAb1427	MBLAb1241	MBLAb1240	MBLAb1239	MBLAb1238	MBLAb1237	MBLAb0561	MBLAb1414	MBLAb1513	-	-	-	-
	**Methanosarcina**	N	23	**1**	1	**1**	1	1	**1**	1	1	1	1	1	1	1	1	1	1	1	0	1	1
	**Other Methanosarcinaceae**	N	38	0.92	1	0.87	1	1	**1**	1	1	1	1	1	1	1	1	1	1	0.67	0.21	0.9	0.67
	**Methanosaetaceae**	N	3	1	1	1	1	1	**1**	1	1	1	1	1	1	1	0.66	1	1	1	0	0	1
**Mbac**	**Methanobrevibacter**	Y	17	1	1	**0.12**	1	1	**0**	1	1	1	1	1	1	1	0.94	1	1	1	1	0.94	1
	**Methanosphaera**	Y	5	1	1	**0**	1	1	**0**	1	1	1	1	1	1	1	1	1	0	0	1	0	0
	**Methanobacterium**	N	9	1	1	**1**	1	1	**1**	1	1	1	1	1	1	1	1	1	1	1	1	1	1
	**Methanothermobacter**	N	3	1	1	**1**	1	1	**1**	1	1	1	1	1	1	1	1	1	1	1	1	1	1
**Mmass**	**Methanomethylophilaceae**	Y	21	**0**	1	**0**	1	0	1	0	0	0	0	0	0	0	0	0	0	0	0	0	0
	**Methanomassiliicoccus intestinalis**	Y	5	**0**	0.93	**0**	1	0	1	0	0	0	0	0	0	0	0	0	0	0	0	0	0
	**Methanomassiliicoccaceae (no M. int.)**	N	6	**1**	1	**0.3**	1	0	1	0	0	0.67	0	0	0	0	0	0	0	0	0	0	0
